# Gut microbial communities from patients with anorexia nervosa do not influence body weight in recipient germ-free mice

**DOI:** 10.1080/19490976.2021.1897216

**Published:** 2021-03-26

**Authors:** Elaine M. Glenny, Farnaz Fouladi, Stephanie A. Thomas, Emily C. Bulik-Sullivan, Quyen Tang, Zorka Djukic, Yesel S. Trillo-Ordonez, Anthony A. Fodor, Lisa M. Tarantino, Cynthia M. Bulik, Ian M. Carroll

**Affiliations:** a Department of Nutrition, Gillings School of Global Public Health, University of North Carolina at Chapel Hill, Chapel Hill, NC, USA; b Department of Bioinformatics and Genomics, University of North Carolina at Charlotte, Charlotte, NC, USA; c Center for Gastrointestinal Biology and Disease, School of Medicine, University of North Carolina at Chapel Hill, Chapel Hill, NC, 27599, USA; Graduate School of Professional Psychology, Morrison Family College of Health, University of St. Thomas, Minneapolis, MN, USA; d Center for Gastrointestinal Biology and Disease, School of Medicine, University of North Carolina at Chapel Hill, Chapel Hill, NC, USA; e Department of Genetics, School of Medicine, University of North Carolina at Chapel Hill, Chapel Hill, NC, USA; f Department of Psychiatry, School of Medicine, University of North Carolina at Chapel Hill, Chapel Hill, NC, USA

**Keywords:** Anorexia nervosa, intestinal microbiota, germ-free/gnotobiotic mice, body weight, adiposity

## Abstract

Anorexia nervosa (AN) is a psychiatric disorder that presents with profound weight dysregulation, metabolic disturbances, and an abnormal composition of gut microbial communities. As the intestinal microbiota can influence host metabolism, the impact of enteric microbial communities from patients with AN on host weight and adiposity was investigated. Germ-free (GF) mice were colonized with fecal microbiotas from either patients with AN (n = 4) prior to inpatient treatment (AN T1, n = 50 recipient mice), the same 4 patients following clinical renourishment (AN T2, n = 53 recipient mice), or age- and sex-matched non-AN controls (n = 4 human donors; non-AN, n = 50 recipient mice). Biological and fecal microbiota data were analyzed with linear mixed-effects models. Body weight did not differ significantly between AN recipient mice (T1 and T2) and non-AN recipient mice following 4 weeks of colonization. Enteric microbiotas from recipient mice colonized with AN T1 and AN T2 fecal microbiotas were more similar to each other compared with enteric microbiotas from non-AN recipient mice. Specific bacterial families in the Actinobacteria, Bacteroidetes, and Firmicutes phyla were significantly associated with body weight, fat mass, and cecum weight irrespective of the donor group. These data suggest that body weight, fat mass, and cecum weight of colonized GF mice are associated with human fecal microbes and independent of donor AN status, although additional analyses with larger cohorts are warranted.

## Introduction

Anorexia nervosa (AN) is a debilitating psychiatric illness that affects 1.4% of women and 0.1% of men in the United States.^1^ Although the pathophysiology of AN remains unclear, a recent genome-wide association study comparing AN cases to healthy controls reported both positive genetic correlations between AN and other psychiatric disorders and negative genetic correlations between AN and metabolic traits, raising the possibility that AN should be reconceptualized as a metabo-psychiatric disorder.^2^ This finding encourages the exploration of potential contributors, in addition to genetic and social factors, to the development and maintenance of AN.

Recent evidence regarding associations between enteric microbes, host metabolism, and host behavior suggests that the intestinal microbiota is a compelling area for exploration.^3–8^ As diet can rapidly change the composition of the intestinal microbiota, depriving enteric microbial communities of nutrients is likely to impact community composition and functions.^9^ Indeed, differences between gut microbiota composition in patients with AN and healthy individuals have been reported by our group and others.^10–17^ Therefore, investigating the functional consequences of AN-associated intestinal microbiotas on weight dysregulation is warranted.

Dysbiosis is a commonly used term to denote a gut microbiota composition that i) is found in individuals with a specific disease and ii) differs from gut microbiotas profiled from healthy individuals.^18^ This term implies the presence of an injurious intestinal microbiota; yet in many cases, the negative consequences of an abnormal gut microbiota have not been demonstrated. It is therefore possible that a disease-associated gut microbiota is compositionally distinct but may not adversely impact the host. An attractive approach to investigate the potential impact of a disease-associated gut microbiota on the host is to transplant uncultured fecal microbiotas into germ-free (GF) mice.^19^ The GF rodent model is powerful as it permits investigators to measure phenotypic changes in the recipient rodent that result as a direct consequence of the transplanted microbial community. Colonizing GF mice with cecal microbiotas from mouse models of diet-induced obesity or genetic hyperphagia is sufficient to increase adiposity in recipient mice as compared with GF mice receiving cecal contents from non-obese controls.^20,21^ Stool samples from obese adult humans transplanted into GF mice have also transmitted obesity-associated phenotypes.^7^ Moreover, Hata et al. recently colonized GF mice with fecal microbiotas from either patients with AN or healthy controls and reported reduced body weight gain and behavioral abnormalities in the offspring of the mice who received AN-associated microbiotas.^22^ However, this study did not include gut microbiota samples from patients following clinical renourishment and weight restoration.

Thus, it is has been demonstrated that prolonged nutrient deprivation in patients with AN alters the intestinal microbiota, and that the AN intestinal microbiota also changes with clinical renourishment. Our study aimed to test whether these abnormal intestinal microbiotas affect body composition and weight gain in recipient GF mice. We also sought to identify microbial taxa in the colonized mice that were associated with body composition, weight gain, and gastrointestinal anatomy.

## Results

### Patients with AN underwent standard treatment and gained significant weight; however, at discharge, patients weighed less than non-AN controls

Patients with AN were admitted to the inpatient unit at the University of North Carolina Center of Excellence for Eating Disorders (UNC CEED) with treatment spanning 4–10 weeks (**Table 1**). Prior to beginning treatment, patients had a mean body weight of 38.1 ± 3.4 kg which was reflected by low body mass indices (BMI; 13.8 ± .8 kg/m^2^) (**Table 1**). Calories were incrementally increased resulting in an average intake of 3,200 kcal/day by discharge (**Table 1**). Patients gained significant body weight with an average increase of 11.5 ± 1.9 kg. Using 18.5–24.9 kg/m^2^ as the standard for a normal BMI range, upon discharge 2 patients had a BMI below 18.5 kg/m^2^ (AN_6: 15.5 kg/m^2^, AN_8: 18.1 kg/m^2^) while the other 2 patients attained a BMI above this threshold (AN_5: 19.5 kg/m^2^, AN_7: 18.9 kg/m^2^) (**Table 1**). Despite gaining substantial weight, the mean body weight for patients at discharge was still significantly lower than the mean body weight for non-AN controls (49.6 ± 3.3 kg *vs*. 60.1 ± 2.6) (**Table 1**).

**Table 1.** **Characteristics of human participants**. *p < .05 unpaired t-test non-AN *vs*. AN T1 and non-AN *vs*. AN T2; ^p < .05 paired t-test AN T1 *vs*. AN T2

ID	Age(years)	T1 weight(kg)	T1 BMI(kg/m^2^)	T1 calories(kcal/day)	T2 weight(kg)	T2 BMI(kg/m^2^)	T2 calories(kcal/day)	Δ weight(kg)	Δ BMI(kg/m^2^)	Δ calories(kcal/day)	Days betweenT1 and T2stool samples
**non-AN_1**	18	54.9	20.4	** **	** **	** **	** **	** **	** **	** **	** **
**non-AN_2**	19	65.9	23.4								
**non-AN_3**	18	62.7	21.2								
**non-AN_4**	19	56.8	22.9								
**Mean±SEM**	**18.5 ± 0.3**	60.1 ± 2.6*	22.0 ± 0.7*								
**AN_5**	18	33.4	13.0	1,200	50.6	19.5	3,600	17.2	6.5	2,400	73
**AN_6**	18	32.6	12.0	800	41.3	15.5	3,200	8.7	3.5	2,400	39
**AN_7**	18	39.3	15.1	1,400	49.2	18.9	2,900	9.9	3.8	1,500	35
**AN_8**	19	47.2	14.9	1,900	57.3	18.1	3,100	10.1	3.2	1,200	31
**Mean±SEM**	**18.3 ± 0.3**	**38.1 ± 3.4**	**13.8 ± 0.8**	**1,325 ± 230**	**49.6 ± 3.3**	**18.0 ± 0.9**	**3,200 ± 150**	**11.5 ± 1.9^**	**4.3 ± 0.8^**	**1,875 ± 300^**	**44.5 ± 9.6**

### Intestinal microbiotas from individuals with AN do not affect weight gain or fat mass accumulation in recipient GF mice

**Figure 1.**
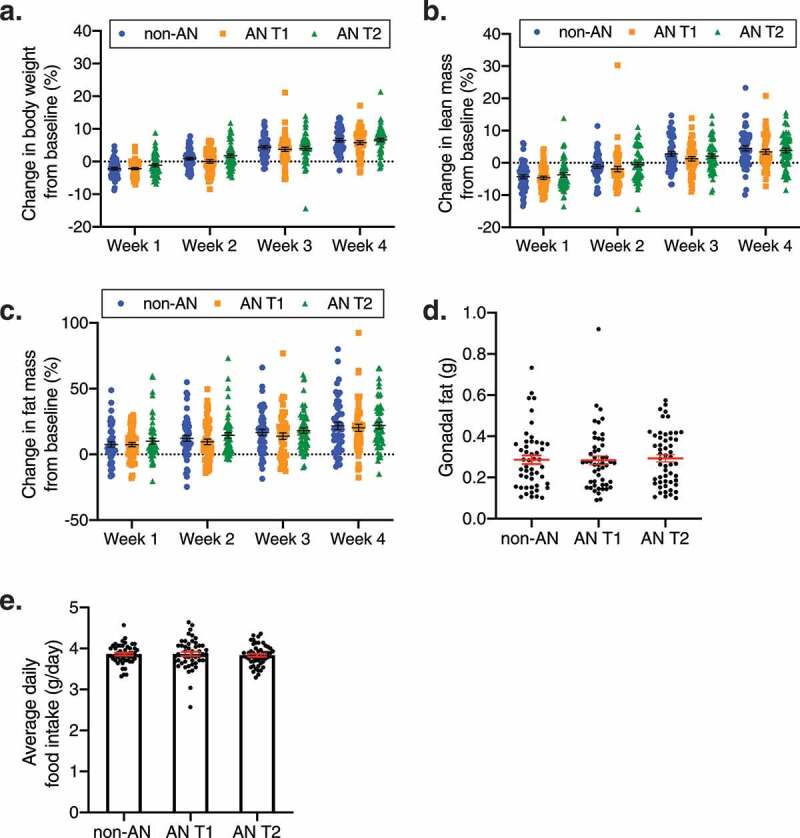
**Body composition in non-AN, AN T1, and AN T2 recipient mice**. (a) Percent change in body weight, (b) lean mass, and (c) fat mass of germ-free mice over the course of 4 weeks colonized with fecal microbiotas from non-AN individuals, patients with AN at time of admission to (AN T1), and patients with AN at time of discharge from (AN T2) the inpatient eating disorder unit. (d) Gonadal fat weight 4 weeks after colonization and (e) average daily food consumption in germ-free mice colonized with fecal microbiotas from non-AN, AN T1, or AN T2 human donors. Mean±SEM

Fecal microbiotas from 4 non-AN individuals collected at a single timepoint and 4 patients with AN collected at 2 timepoints (admission to an inpatient eating disorder unit [AN T1] and upon discharge from the unit [AN T2]) were transplanted into 153 GF mice (n = 50 non-AN controls, n = 50 AN T1, n = 53 AN T2). Since the number of mice was greater than the number of independent donors in our dataset we used linear mixed-effects models with a random term for human donor identity. Following 4 weeks of colonization, there were no significant body weight gain differences in GF mice that received AN T1 fecal microbiotas compared with GF mice that received non-AN fecal microbiotas (adjusted *p* = .24) (Figure 1a). This observation did not change when investigating sex as a biological variable (male adjusted *p* = .66; female adjusted *p* = .78, Supplementary Figure 1a-b). Consistent with a previous report, weight gain was observed in all mouse groups between weeks 2 and 3 (Figure 1a).^23^ Additionally, we observed no significant difference in body weight change between AN T1 or AN T2 recipient mice (Figure 1a), and this finding was also not influenced by sex (Supplementary Figure 1a-b). GF mice colonized with non-AN, AN T1, and AN T2 fecal microbiotas also did not exhibit significant differences across groups for fat or lean mass accumulation or daily food consumption (Figure 1b**-e,** Supplementary Figure 2**)**.

### Cecum weights did not differ significantly between recipient GF mice colonized with intestinal microbiotas from individuals with AN compared with recipient GF mice colonized with intestinal microbiotas from non-AN controls

**Figure 2.**
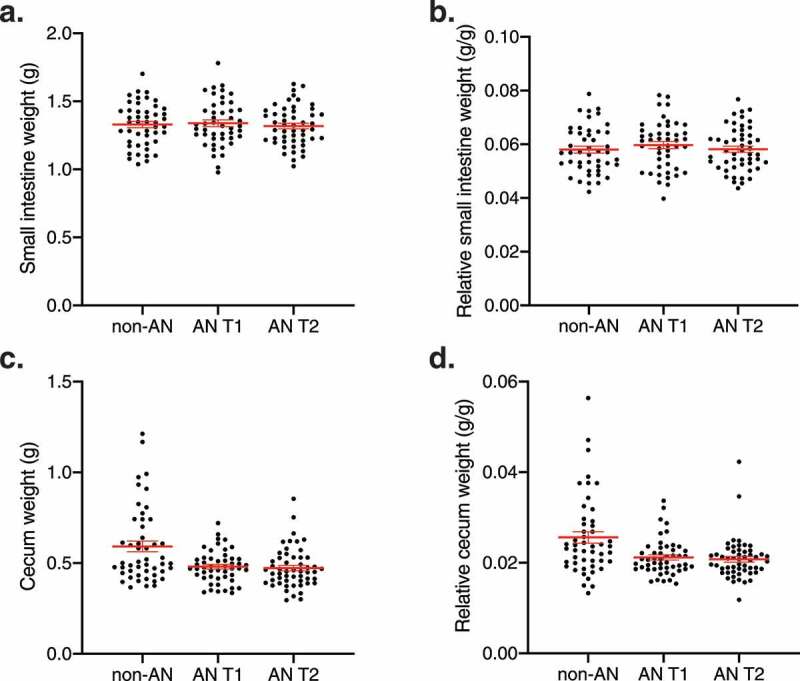
**Small intestinal and cecum weights in non-AN and AN (T1 & T2) recipient mice**. (a) Small intestine weight, (b) relative small intestine weight, (c) cecum weight, and (d) relative cecum weight of germ-free mice colonized with fecal microbiotas from non-AN individuals, patients with AN at time of admission to (AN T1), and patients with AN at time of discharge from (AN T2) the inpatient eating disorder unit. Relative small intestine weight and relative cecum weight are defined as gram of tissue per gram of mouse body weight at time of euthanasia. Mean±SEM

Given that intestinal microbial communities are in direct contact and constantly interact with the gastrointestinal tract, we investigated whether an AN-associated gut microbiota influenced the gross intestinal anatomy of recipient mice. We found no changes in the weight of the small intestines across groups (adjusted *p* = .94) (Figure 2a**-**b). Similarly, ceca from AN T1 and AN T2 recipient mice did not differ in weight compared with ceca from non-AN recipient mice (non-AN *vs*. AN T1 adjusted *p* = .09, non-AN *vs*. AN T2 adjusted *p* = .09, AN T1 *vs*. AN T2 adjusted *p* = .91, Figure 2c**-**d). These findings were also not influenced by the sex of the recipient mice (Supplementary Figure 3).

### Composition of fecal microbiotas from AN T1 and AN T2 recipient mice are more similar to each other than to fecal microbiotas from non-AN recipient mice

**Figure 3.**
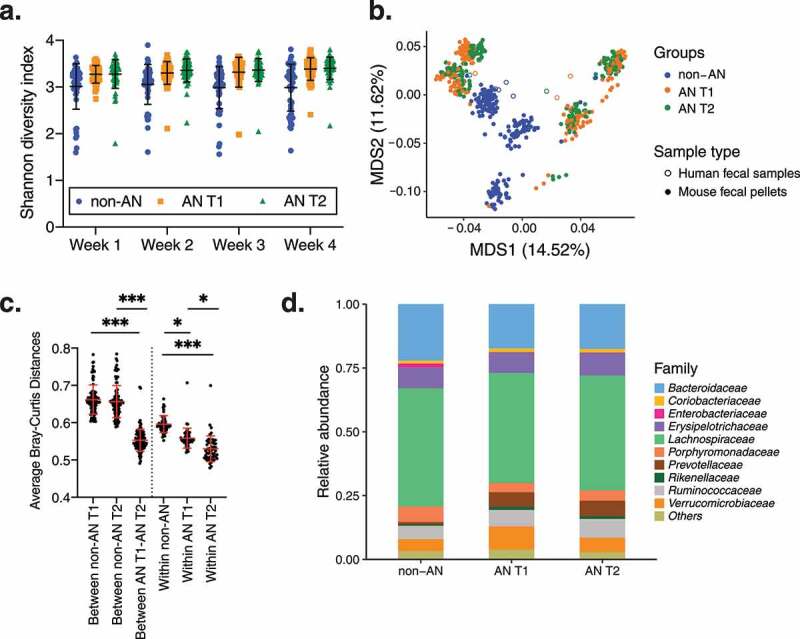
**Diversity between and within fecal microbial communities in colonized mice**. (a) Shannon diversity of fecal pellets from mice colonized with fecal microbiotas from non-AN individuals, patients with AN at time of admission to (AN T1), and patients with AN at time of discharge from (AN T2) the inpatient eating disorder unit. (b) Multi-dimensional analysis of donor stool samples and all fecal samples collected from recipient germ-free mice colonized with fecal microbiotas from non-AN controls and patients with AN (AN T1 and AN T2). (c) Average Bray–Curtis distances within and between groups. (d) Average microbial taxonomic profile at the family level from the fecal pellets of mice colonized with fecal microbiotas from non-AN individuals, patients with AN at time of admission to (AN T1), and patients with AN at time of discharge from (AN T2) the inpatient eating disorder unit at week 4 following colonization. Mean±SD. *adjusted *p*<.05, **adjusted *p*<.01, ***adjusted *p*<.001

We next investigated whether the intestinal communities of these colonized mice were compositionally different across groups. Irrespective of the sex of the recipient mouse, the Shannon diversity index–a measure of community richness–did not differ significantly between microbiotas from AN T1 and AN T2 recipient mice compared with non-AN recipient mice at all 4 weeks following colonization (non-AN *vs*. AN T1 and non-AN *vs*. AN T2 adjusted *p* = .09, AN T1 *vs*. AN T2 adjusted *p* = .84, time *p* = .06, Figure 3a, Supplementary Figure 4). Multi-dimensional scaling (MDS) analysis using Bray–Curtis dissimilarity indices revealed that microbial communities from AN T1 and AN T2 recipient mice were also not distinct from each other across MDS1 and MDS2 (non-AN *vs*. AN T1 and non-AN *vs*. AN T2 across MDS1 adjusted *p* = .86, non-AN *vs*. AN T1 and non-AN *vs*. AN T2 across MDS2 adjusted *p* = .10, Figure 3b **and**Supplementary Figure 5a-c). However, microbial communities were distinct between non-AN and AN T1 and between non-AN and AN T2 recipient male mice across MDS2 (adjusted *p* = .019 Supplementary Figure 5d). As expected, fecal microbiotas from mice colonized with stool from the same AN donor (i.e., matched AN T1 and AN T2) clustered together (Supplementary Figure 5e). Interestingly, fecal microbiotas from mice colonized with stool from donors who did (n = 2; AN_5 and AN_7) or did not (n = 2; AN_6 and AN_8) achieve a BMI above 18.5 kg/m^2^ at the time of discharge also clustered together.

We further compared average Bray–Curtis distances at week 4 post-colonization. Average Bray–Curtis distances revealed more similarity (i.e., significantly shorter distances) between fecal microbiotas from AN T1 and AN T2 recipient mice compared with fecal microbiotas from non-AN recipient mice (average Bray–Curtis distances between non-AN and AN T1 = .66 ± .04; between non-AN and AN T2 = .66 ± .04; between AN T1 and AN T2 = .55 ± .03 adjusted *p* < .001, Figure 3c). In addition, average Bray–Curtis distances within the non-AN group were significantly greater than the average Bray–Curtis distances within AN T1 and AN T2 groups (within non-AN *vs*. within AN T1 adjusted *p* = .02 and within non-AN *vs*. within AN T2 adjusted *p* = .001). Average Bray–Curtis distances within the AN T1 recipient group were also significantly greater than distances within the AN T2 recipient group (adjusted *p* = .045). These results confirm that fecal microbiotas of AN T1 and AN T2 recipient mice are more similar to each other than to the microbiotas of non-AN recipient mice. Given the overall differences in microbiotas between AN and non-AN recipient mice, we also investigated which specific bacterial taxa were driving these differences; however, we found that the taxonomic profiles of fecal microbiotas from AN T1 and AN T2 recipient mice were not significantly different compared with non-AN recipient mice at week 4 post-colonization (Figure 3d and **Supplementary** Figure 6), irrespective of recipient mouse sex.

### Colonization efficiencies are not different between intestinal microbiotas from patients with AN and non-AN individuals

**Figure 4.**
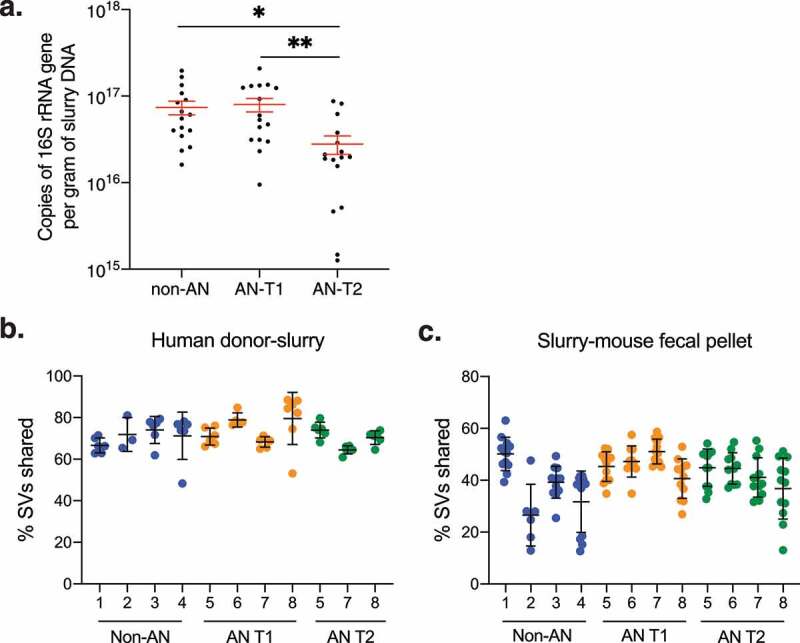
**Bacterial load and engraftment rate of AN-patient and non-AN fecal slurries and recipient mice**. (a) Copies of 16S rRNA genes per gram of DNA in donor fecal slurries. Mean±SEM. (b) Percent shared sequence variants (SVs) between donor samples and fecal slurries between patients with AN and non-AN donor groups. Mean±SD. (c) Percent shared sequence variants (SVs) between fecal slurries and recipient mice between patients with AN and non-AN donor groups. Mean±SD. *p<.05, **p<.01

As collection protocols dictated different handling conditions for fecal samples from non-AN participants and patients with AN, we sought to determine if either the microbial load in fecal slurries or the efficiency of microbial engraftment into mice differed between experimental groups. Quantitative PCR (qPCR) of the 16S rRNA gene revealed that the bacterial load was lower than AN T2 in fecal slurries compared with non-AN and AN T1 fecal slurries (Figure 4a). To investigate microbial engraftment efficiency in colonized mice we used previously described equations^24^ and found that the percent of shared sequence variants (SVs) between donor samples and fecal slurries or between donor fecal slurries and recipient mice did not significantly differ between non-AN, AN T1, or AN T2 experimental groups (Figure 4b-c). These findings suggest that the technical differences in fecal sample collection and storage between human donor groups did not have a meaningful impact on slurry microbial mass or ultimately colonization of recipient mice.

### Specific bacterial families from human donor stool are associated with cecum weight and changes in body weight and fat mass of colonized GF mice

**Figure 5.**
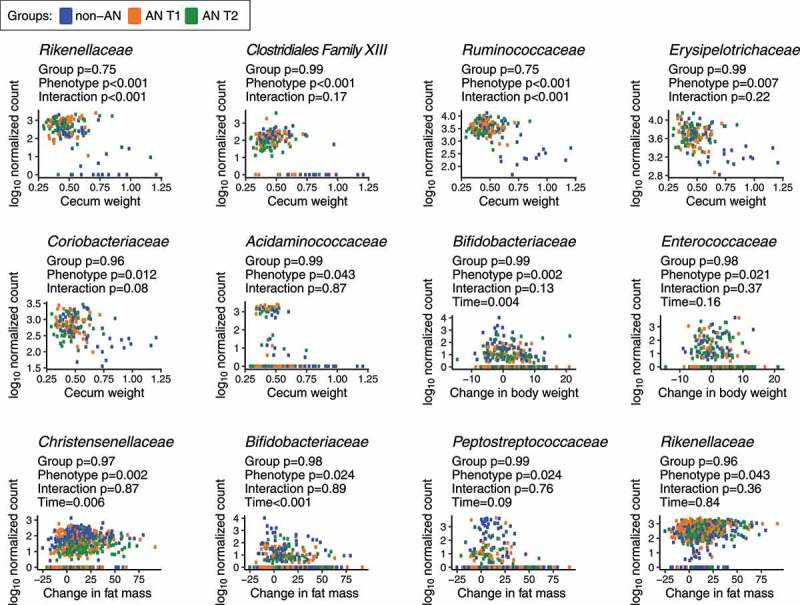
**Bacterial families associated with physiological changes in non-AN and AN (T1 & T2) recipient mice**. *p*-values shown are from a linear mixed-effects model with log_10_-normalized bacterial taxa as the dependent variable; groups, study outcomes, group and study outcome interactions, and time as fixed effects; and donors as random effects. *p*-values were adjusted using the Benjamini-Hochberg procedure at a 5% false discovery rate (FDR)

Finally, we interrogated whether specific bacterial families were associated with our principal study outcomes of daily food consumption, change in body weight, change in fat and/or lean mass, gonadal fat weight, small intestine weight, and cecum weight. We constructed linear mixed-effects models with log_10_-normalized bacterial taxa as the dependent variable; groups, study outcomes, group and study outcome interactions, and time as fixed effects; and donors as random effects. Under this model, we observed that 6 out of 26 non-rare bacterial families were significantly associated with cecum weight, including *Rikenellaceae, Clostridiales Family XIII, Ruminococcaceae, Erysipelotrichaceae, Coriobacteriaceae, and Acidaminococcaceae* (**Figure 5**). Interestingly, among these families, associations between *Rikenellaceae* and *Ruminococcaceae* and cecum weight were modified by the human donor group (adjusted *p* < .05). The families *Christensenellaceae, Bifidobacteriaceae, Peptostreptococcaceae, Alcaligenaceae, Enterococcaceae, and Rikenellaceae* were significantly correlated with changes in fat mass accumulation or body weight gain in AN and non-AN colonized mice (adjusted *p* < .05); however, the associations between these families and body weight or fat mass were independent of the human donor group. We conclude that multiple taxa were robustly associated with weight phenotypes in our mouse models, although the connection to the AN versus the non-AN status of the donors was more tenuous.

## Discussion

As established guidelines for treating patients recovering from AN have failed to curtail either the high mortality or the relapse rate of this pernicious illness, we investigated the role of a novel contributor, the intestinal microbiota, that may influence features of AN and could potentially inform novel approaches to treatment.^25^ Support for this idea comes from the established association between the gut microbiota and adiposity, and emerging reports of enteric microbes influencing host behavior.^3–8^ Ultimately, understanding the role(s) that intestinal microbial communities play in the progression of or recovery from AN could provide new mechanistic insights into this perplexing illness and guide new treatment paradigms.

We observed no relationship between AN-associated intestinal microbiotas and changes in body weight, fat mass, or lean mass at any timepoint in recipient mice. These observations suggest that enteric microbes harbored in patients with AN may not influence host adiposity or body weight. Our findings are in contrast with a recent study published by Hata and colleagues that reported a decreased rate of weight gain in GF mice colonized with fecal microbiotas from patients with AN compared with GF mice colonized with fecal microbiotas from healthy controls.^22^ Our current study differs from this published study in numerous ways, the sum total of which may largely account for the major discrepancies found between these two reports. Firstly, and perhaps most importantly, Hata et al.^22^ colonized GF breeders with fresh (i.e., never frozen) human stool and subsequently measured body weight gain of the gnotobiotic female offspring from 4 to 10 weeks of age (i.e., weaning until adulthood). In contrast, we colonized adult 8- to 9-week-old GF female and male mice with slurries from freshly thawed human stool and followed weight gain and fat and lean mass trajectories for 4 weeks. Therefore, the major shared outcome between these two studies–body weight gain over time–was measured at two very different life stages in the rodents. Specifically, 4- to 10-week-old mice are in a substantial period of growth and weight gain, while the rate of body weight gain is curtailed in adult mice.

While transplanting human feces directly into GF mice is currently the most widely adopted approach in rodent colonization studies, colonizing breeders with fresh human feces and studying the resulting offspring is an interesting idea that should be further explored.^3,7,26–28^ However, given that pregnancy is known to change microbial communities in dams,^29^ the percent resemblance between the gut microbiotas of the offspring and the original human donor is critical to report.^24^ Any epigenetic or behavioral changes in the offspring resulting purely from pregnant dams harboring distinct microbial communities are potentially important confounders.^27,30^ Additionally, these two studies used mice on two different genetic backgrounds–Hata and colleagues used BALB/c mice whereas we used C57BL/6 mice.

Finally, similar to our study design, Hata et al. used more mice than human donors but analyzed their data using ANOVA models rather than linear mixed-effects models. ANOVAs potentially violate assumptions of independence as these models may overstate significance if the microbial community composition of mice is dependent on donor identity. Our study, therefore, used a more appropriate biostatistical approach (linear mixed-effects models) to determine whether the phenotypes in recipient mice were dependent on the donor from which the microbial communities originated. Given the importance of establishing the contribution of the intestinal microbiota in AN, future studies are necessary to replicate either the positive results of Hata et al. or the largely negative results we report here.

Despite no differences in cecum weight between recipient mouse groups, the bacterial families *Rikenellaceae* and *Ruminococcaceae* were significantly associated with cecum weight depending on the human donor. These results further suggest that AN-associated gut microbiotas and their metabolites produced within the cecum may affect cecum physiology differently from non-AN microbiotas. However, the translational relevance of this finding is difficult to discern as the cecum in the mouse functions as a site for microbial fermentation while the cecum in humans has little fermentative capacity.^31^ Nonetheless, as GF mice both lack microbes for fermentation and exhibit enlarged ceca, it is tempting to speculate that AN-associated gut microbiotas are rich in microbes with elevated fermentative capacities.^32^ Indeed, the *Rikenellaceae* family (enriched in the AN-colonized mice in our study) outcompetes other common digestive tract bacteria when grown on mucin-rich media.^33^ Additionally, the *Ruminococcaceae* family (also enriched in the AN-colonized mice in our study) encompasses microbes known to produce short-chain fatty acids (SCFA) *via* fermentation of fiber (i.e., *Faecalibacterium prausnitzii* and *Clostridium leptum*).^34,35^ It has yet to be elucidated whether SCFAs play a role in AN and whether SCFA production is a compensatory response to a nutrient-deprived environment.

We recently reported that fecal slurries adequately represent the microbial composition of human donor fecal samples.^24^ Although fecal slurries mirror human donor samples, only 42% of microbes successfully transfer from human stool samples into recipient GF mice, and the microbes classed within the Firmicutes phylum exhibit a particularly low transfer rate.^24^ It is possible that the approach we used to collect, store, and prepare fecal samples for colonization could have influenced this transfer rate; however, other studies that have used alternative approaches to protect fecal microbes from oxygen (e.g., storage in pre-reduced glycerol or N_2_ gas) prior to colonizing GF recipient mice report similar engraftment rates as our study.^36,37^ Moreover, colonization of GF mice with fresh or frozen feces from the same human donor exhibit similar microbial communities.^21^ In our present study, we found that the colonization rate in recipient mice did not differ between patients with AN and non-AN donor groups irrespective of the disparate collection approaches. Given these relatively low colonization rates, it is feasible that adiposity-modulating enteric microbes within patients with AN may have failed to colonize and were consequently unable to influence physiological outcomes in recipient mice. Thus, patients with AN may harbor adiposity-regulating enteric microbes but transplanting uncultured human stool samples into GF mice may not identify these microorganisms. Identification of specific microorganisms of interest followed by monocolonization experiments could overcome this limitation and may be a more fruitful approach for future studies. However, GF mice have an underdeveloped immune system^38^ and transplanting fecal microbiotas from patients with AN, or a single microorganism, into GF mice will initiate a series of novel immune interactions which may result in enteric microbes being incapable of influencing adiposity in recipient mice.

In conlusion, we found that microbiotas from AN donors did not influence weight gain or body composition in recipient mice. Although we did not identify specific bacterial strains that influence adiposity in recipient mice, the bacterial families we highlight in this study provide a foundation for future gnotobiotic studies investigating the influence of specific members of the intestinal microbiota on host adiposity. Additionally, future experiments to test whether metabolic phenotypes would emerge if mice were maintained on either a food-restricted diet or a diet to better mimic clinical renourishment protocols and subsequently transplanted with fecal microbiotas from patients with AN are warranted. Moreover, given the established relationship between diet and the gut microbiota,^9^ developing a mouse diet that mirrors the nutritional and caloric contents of patients with AN during renourishment may yield promising results. Future studies should also increase the number of human donors to elucidate the contribution, and the effect size of this contribution, of AN-associated microbial communities on host body composition and metabolism.

## Methods

### Human subjects

Stool samples came from a clinical trial registered at ClinicalTrials.gov (Identifier: NCT03119272). The Biomedical Institutional Review Board (University of North Carolina [UNC] at Chapel Hill) approval was obtained for the collection of human stool samples. Written consent was also obtained from all participants prior to study participation.

Data from adult female patients with AN (n = 4) and age- and sex-matched non-AN controls (n = 4) were collected for this study. Eligible inpatient participants were evaluated by trained professionals at the UNC Center of Excellence for Eating Disorders (CEED) and met DSM-5 criteria for AN.^39^ Recruitment of non-AN controls occurred through university flyers and listservs. Non-AN controls had no history of either a body mass index (BMI) outside 18.5–24.9 kg/m^2^ or any eating disorder. Exclusion criteria for all study participants were based on factors that influence the composition of gut microbial communities, including the history of gastrointestinal tract surgery (other than cholecystectomy) or any clinical diagnosis that could explain chronic or recurring bowel symptoms (e.g., inflammatory bowel diseases, irritable bowel syndrome, or celiac disease, treatment in the previous 2 months with antibiotics, nonsteroidal anti-inflammatory drugs, or steroids, and intentional use of probiotics in the previous 2 months).

### Human stool sample collection

Our protocol for collecting stool samples from patients with AN and non-AN controls has been previously described.^12^ Briefly, patients with AN provided a stool sample at admission to CEED (AN T1) and discharge from CEED after clinical renourishment and weight restoration (AN T2). Non-AN controls provided a single stool sample. All samples were stored at 4 °C until they were either transported (AN samples) or shipped overnight with ice packs (non-AN samples) to our laboratory. Upon arrival to the laboratory, fresh stool samples were immediately mechanically homogenized, aliquoted into 2-mL cryovials, and stored at −80 °C until needed for transplantation into GF mice.

### Colonization of GF mice with human fecal microbiotas

UNC Institutional Animal Care and Use Committee approval was obtained prior to animal studies being conducted. Freshly thawed human feces were prepared in an anaerobic chamber using 15 mL screw-top tubes containing prereduced phosphate-buffered saline (10% w/v) to create fecal slurries under anaerobic conditions.^21^ Slurries were vortexed for 5 min and then centrifuged (1,000 × g for 3 min or 9 × g for 3 min) or filtered (100 μm filter) to separate suspended bacteria from fibrous fecal material. Filtered slurries were then aliquoted (300 μL) into individual 2 mL cryotubes. 8- to 9-week-old adult male and female C57BL/6 GF mice were randomized into blinded groups and orally gavaged with 10 mL/kg (250 μL maximum) of the prepared slurry.

### Mouse husbandry and tissue harvest

Mice were singly housed in individually ventilated cages with *ad libitum* access to autoclaved water and rodent chow (2020SX, Teklad) to avoid cage effects on the enteric microbiotas.^40^ Fresh fecal pellets, body weights, and food consumption were collected every 7 days. Body composition was also measured every 7 days using a dual-energy X-ray absorptiometry (DEXA) machine (Lunar PIXImus, GE Lunar Corporation, WI, USA). 4 weeks after colonization, mice were euthanized with CO_2_ followed by cervical dislocation. To investigate the influence of gut microbiotas from human donors on the host, the gonadal fat pads, the cecum, and the small intestine were excised and weighed.

### 16S rRNA gene sequencing and data processing

DNA extraction from human and mouse fecal samples were performed using a combination of physical disruption of bacterial cells and phenol-chloroform extraction, followed by a DNA clean-up kit (Qiagen DNeasy Blood and Tissue extraction kit, Valencia, CA) as previously described.^41^ Fecal microbiotas were characterized using polymerase chain reaction (PCR) to create variable 4 (V4) region 16S rRNA gene (515–806 bp) libraries for sequencing on the Illumina MiSeq platform (Illumina, San Diego, CA) at the High-Throughput Sequencing Facility in the Carolina Center for Genome Sciences at the UNC School of Medicine as previously described.^13^

Raw sequences were processed as described in Fouladi et al.^24^ Briefly, sequences were demultiplexed by an automatic bioinformatics pipeline “BioLockJ” (https://github.com/BioLockJ-Dev-Team/BioLockJ). Exact amplicon sequence variants (SVs) were generated by the DADA2 pipeline and taxonomic classification was performed using a DADA2-formatted reference database (silva_nr_v128_train_set.fa.gz) and “assignTaxonomy*”* function from DADA2.^42^ To control for differences in sequencing depths between samples, count data were normalized using the formula described.^24^

### Quantitative PCR (qPCR)

qPCR was performed using DNA extracted from 250 μl of donor fecal slurries with primers that amplify the 16S rRNA gene as previously described.^43^ Briefly, qPCR assays were conducted in 384-well plates on a real-time CFX 384 Real-Time System (BioRad, Hercules, CA). Each PCR was carried out in a final volume of 25 μl and contained the following: 1 × SYBR Select Master Mix (Applied Biosystems, Foster City, CA), 0.2 μM of each primer and 40 ng of purified fecal DNA. PCR conditions were as follows: 10 min at 95°C, followed by 40 cycles of 95°C for 15 seconds, 50°C for 20 seconds, and 72°C for 20 seconds. Melt curve analysis of PCR products was conducted following each assay to confirm that the fluorescent signal originated from specific PCR products and not from primer-dimers or other artifacts. Absolute number of 16S rRNA gene copies were calculated using a standard curve generated from known concentrations of microbial DNA and data were normalized per gram of slurry DNA.

### Statistical analyses

Linear mixed-effects models with recipient mouse groups and time as fixed effects and human donors as random effects were used to compare the percent change in body weight, percent change in fat mass, percent change in lean mass, average daily food consumption between groups. In cases where study outcomes were compared across groups at a single time point (i.e., gonadal fat weight, small intestinal weight, and cecum weight at week 4 or changes in body weight at each time point), a linear mixed-effects model without time as a fixed effect was used. For all linear mixed-effects models, the “summary” function in R was used for pairwise comparisons between non-AN, AN T1, AN T2 groups, and *p*-values were adjusted using the Benjamini–Hochberg procedure at a 5% false discovery rate (FDR).^44^

Shannon diversity index was used as a measurement of α-diversity and was calculated through the “diversity” function from the vegan package in R (version 2.5–6). Multi-dimensional scaling (MDS) analysis of Bray–Curtis dissimilarity indices was used to visualize the dissimilarity in the microbial communities between fecal samples. “capscale” and “vegdist” functions from the vegan package in R were used for analyses of MDS and Bray–Curtis dissimilarity indices, respectively. The Shannon diversity index and microbial composition of recipient mice across the first and second axes of MDS (MDS1 and MDS2) were compared between groups using a linear mixed-effects model (with recipient mouse groups and time as fixed effects and human donors as random effects). Average Bray–Curtis distances at week 4 post-colonization were compared between and within groups using a linear mixed-effects model. Similar to analyses of phenotypes, the “summary” function in R was used for pairwise comparisons between non-AN, AN T1, AN T2 groups and *p*-values were adjusted using the Benjamini–Hochberg procedure at a 5% false discovery rate (FDR).

The log_10_ normalized count of each bacterial taxon that was present in at least 10% of mouse fecal pellets was compared between recipient GF mice colonized with microbiotas of non-AN controls, patients with AN at admission to the inpatient eating disorder unit (AN T1), and patients with AN at discharge from the inpatient eating disorder unit (AN T2) using a linear mixed-effects model with human donors as random effects (taxa ~ group + time, random = ~1 | donor). Finally, for each taxon that was present in at least 10% of mouse fecal pellets, a linear mixed-effects model was constructed with bacterial taxa as the dependent variable; groups, study outcomes, group and study outcome interactions, and time as fixed effects; and donors as random effects (taxa ~ group * phenotype + time, random = ~1 | donor). These linear mixed-effects models were used to determine if taxa transferred from a donor group were associated with a study outcome in recipient mice. *p*-values were adjusted for multiple hypothesis testing using a 5% FDR with the Benjamini-Hochberg procedure (the number of tests is equal to the number of non-rare taxa examined). Finally, qPCR data were analyzed using a one-way ANOVA followed by a Tukey’s post-hoc test.

## Supplementary Material

Supplemental Material

## Data Availability

The datasets generated and analyzed during the current study are available in the NCBI Sequence Read Archive (PRJNA558494), and all scripts are available at https://github.com/FarnazFouladi/AnorexiaMicrobiota.git.
