# Supplementary material for: Gut microbial communities from patients with anorexia nervosa do not influence body weight in recipient germ-free mice
Source: Gut Microbes. 2021 Mar 26;13(1):1897216. doi: 10.1080/19490976.2021.1897216 (PMC8007138; doi:10.1080/19490976.2021.1897216)
Supplement: Supplemental Material [file KGMI_A_1897216_SM5534.zip › Document.rtf]

Supplementary Figure 1. Body weight percent change in male and female recipeient mice colonized with non-AN, AN T1, and AN T2 fecal microbiotas. Percent change in body weight in (A) female and (B) male mice. Mean±SEM.
Supplementary Figure 2. Percent change in fat and lean mass in male and female recipeient mice colonized with non-AN, AN T1, and AN T2 fecal microbiotas. Percent change in fat mass in (A) female and (B) male mice. Percent change in lean mass in (C) female and (D) male mice. Gonadal fat mass in (E) female and (F) male mice. Average daily food intake in (G) female and (H) male mice. Mean±SEM. 
Supplementary Figure 3. Small intestine and cecum weights in male and female recipient mice colonized with non-AN, AN T1, and AN T2 fecal microbiotas. Cecum weight in (A) female and (B) male mice and relative cecum weight in (C) female and (D) male mice. Small intestine weight in (E) female and (F) male mice and relative small intestine weight in (G) female and (H) male mice. Relative small intestine weight and relative cecum weight is defined as gram of tissue per gram of mouse body weight at time of euthanasia. Mean±SEM.
Supplementary Figure 4. Diversity within fecal microbial communities in male and female colonized mice. Shannon diversity of fecal pellets from (A) female and (B) male mice colonized with fecal microbiotas from non-AN individuals, patients with AN at time of admission to (AN T1), and patients with AN at time of discharge from (AN T2) the inpatient eating disorders unit. Mean±SD.
Supplementary Figure 5. Diversity between fecal microbial communities in male and female colonized mice. MDS1 of fecal pellets from (A) female and (B) male mice colonized with fecal microbiotas from non-AN individuals, patients with AN at time of admission to (AN T1), and patients with AN at time of discharge from (AN T2) the inpatient eating disorders unit. MDS2 of fecal pellets from (C) female and (D) male mice colonized with fecal microbiotas from non-AN individuals and AN T1 and T2 patients. (E) Multi-dimensional analysis of donor stool samples and all fecal samples collected from recipient germ-free mice colonized with fecal microbiotas from non-AN controls and patients with AN (AN T1 and AN T2) colored by donor. Mean±SD.

Supplementary Figure 6. Taxonomy differences between fecal microbial communities in colonized mice. Average microbial taxonomic profile at the genus level in fecal pellets from mice colonized with fecal microbiotas from non-AN individuals, patients with AN at time of admission to (AN T1), and patients with AN at time of discharge from (AN T2) the inpatient eating disorders unit at week four following colonization.
