# Supplementary figures and images for: Gut microbial communities from patients with anorexia nervosa do not influence body weight in recipient germ-free mice
Source: Gut Microbes. 2021 Mar 26;13(1):1897216. doi: 10.1080/19490976.2021.1897216 (PMC8007138; doi:10.1080/19490976.2021.1897216)

**A.** Females

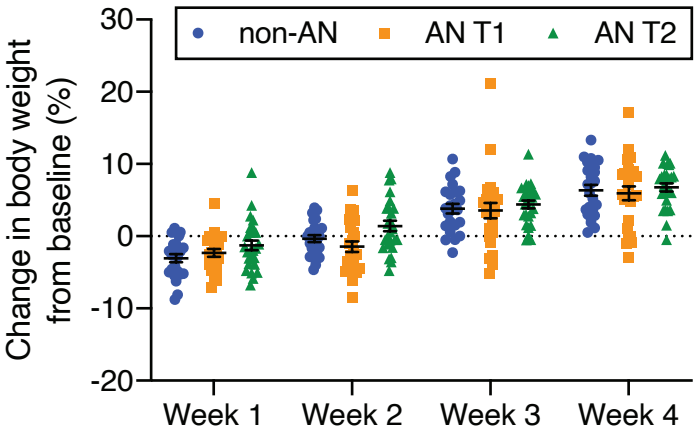

**B.** Males

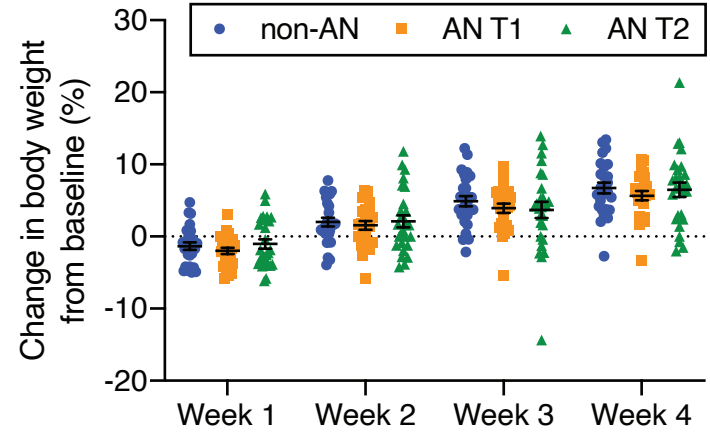

Supplement: Supplemental Material [file KGMI_A_1897216_SM5534.zip › SuppFig1.pdf]

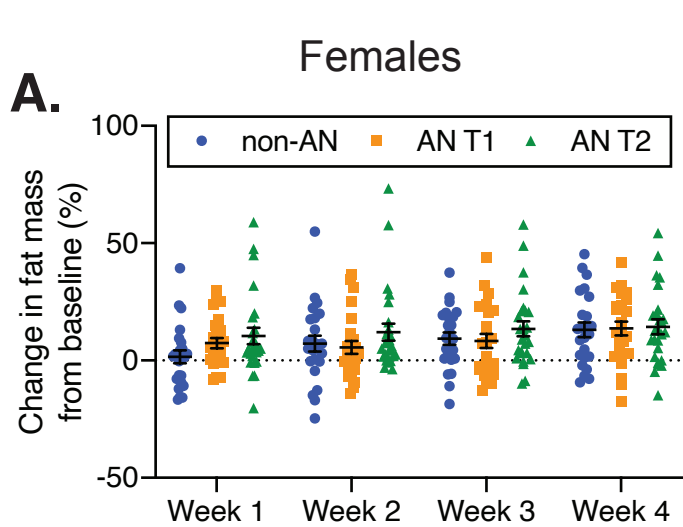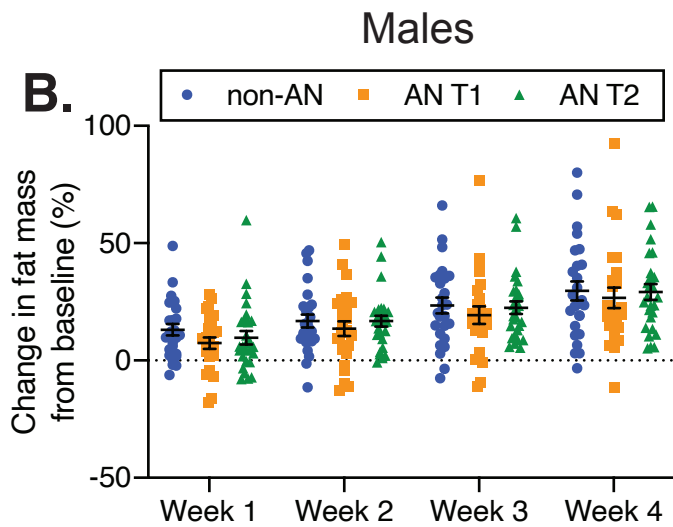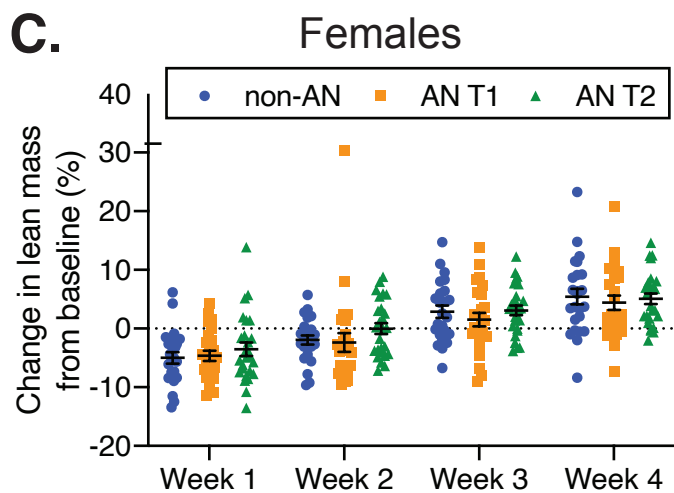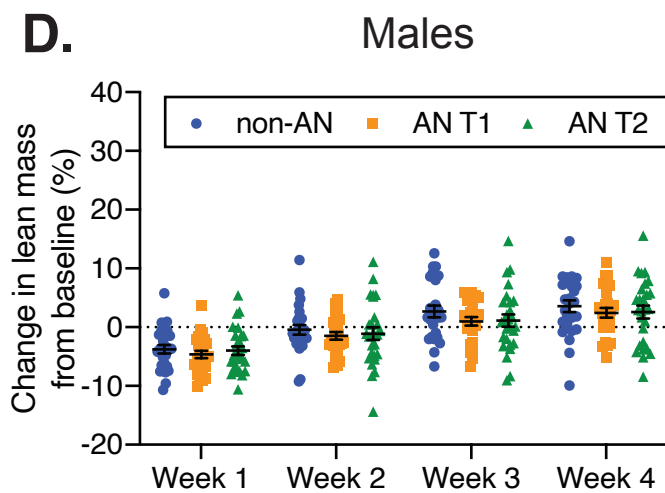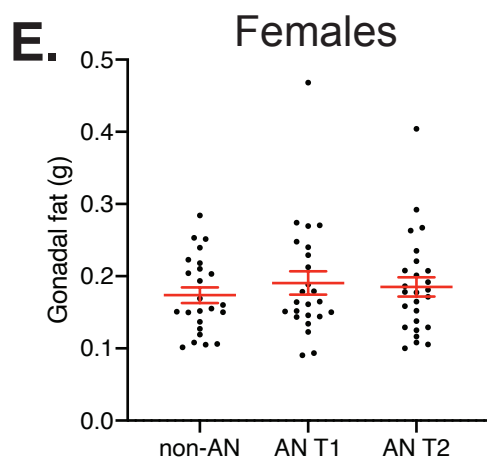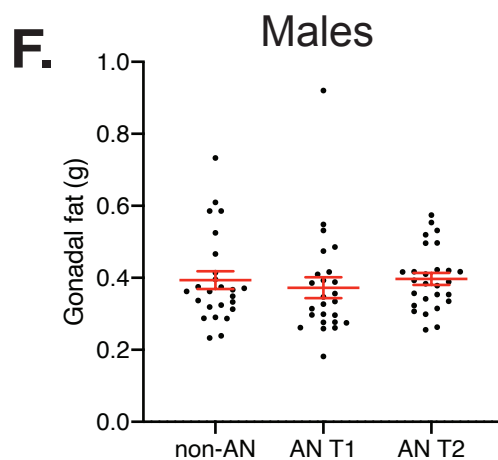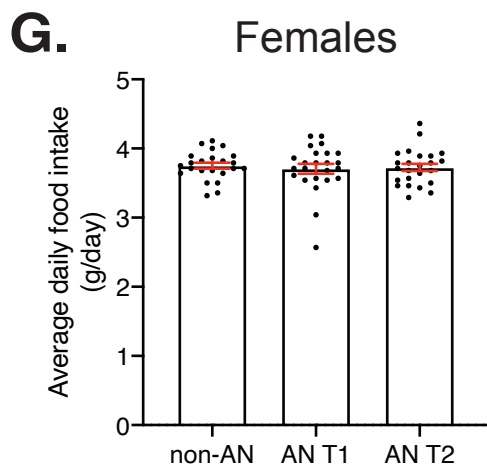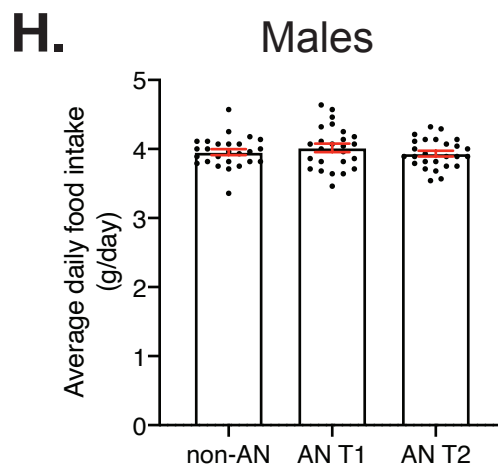

Supplement: Supplemental Material [file KGMI_A_1897216_SM5534.zip › SuppFig2.pdf]

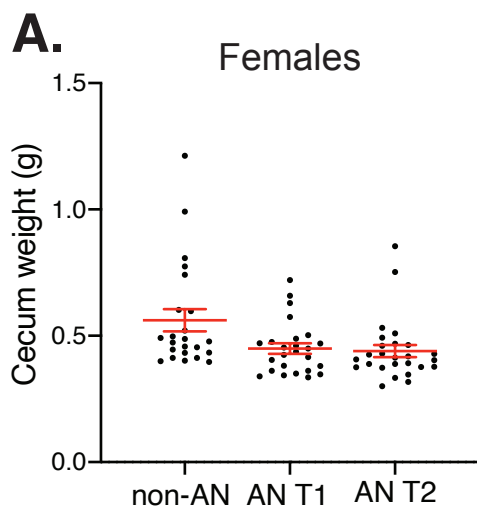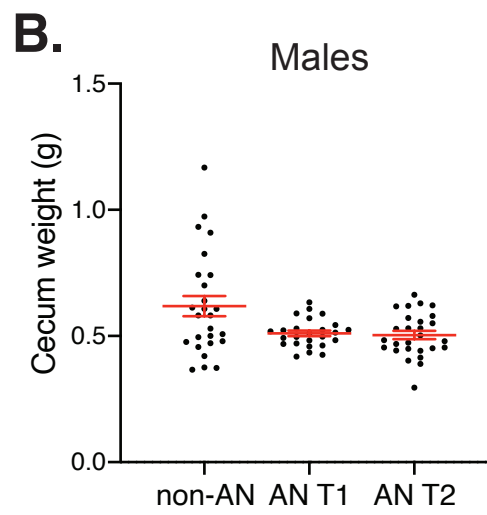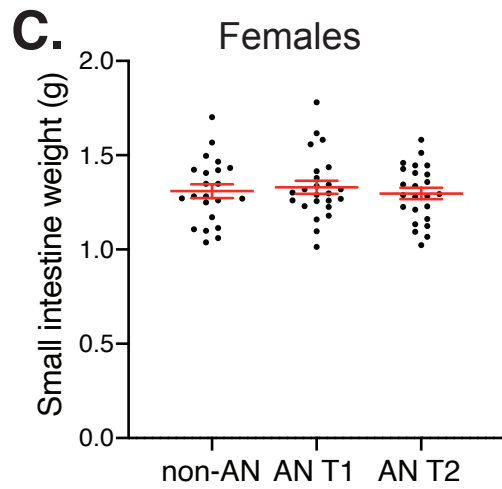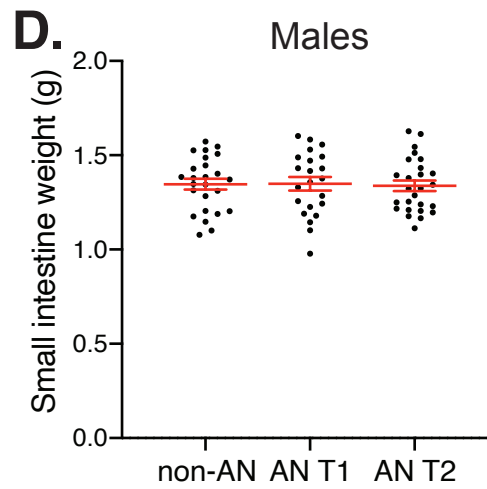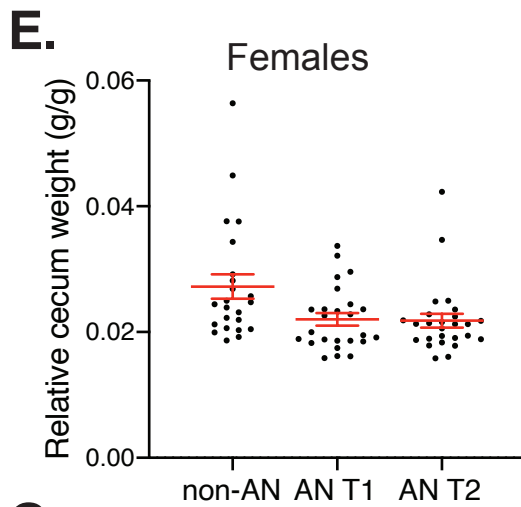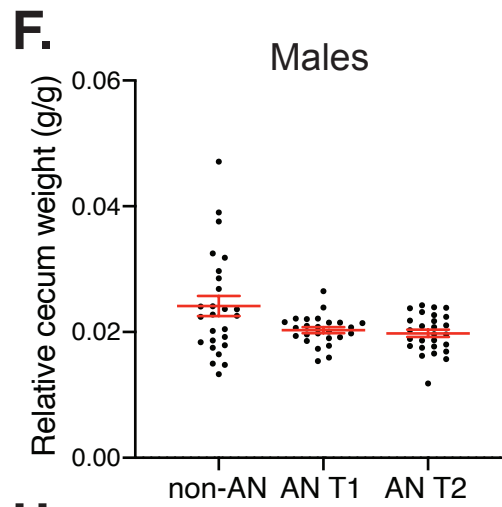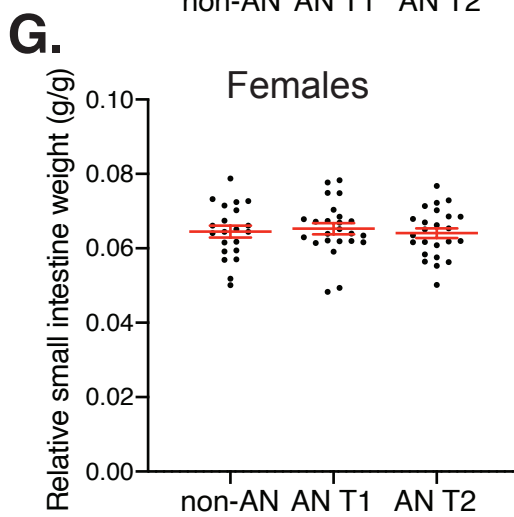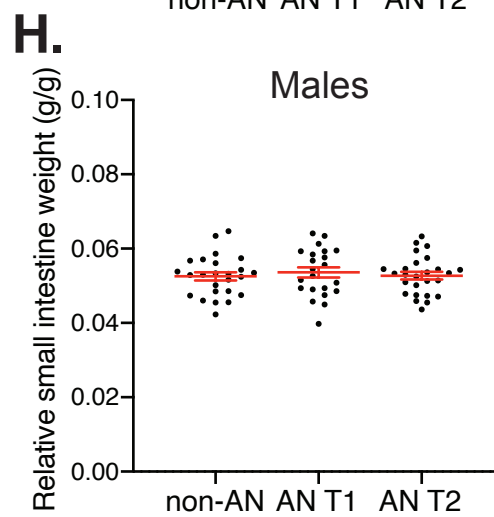

Supplement: Supplemental Material [file KGMI_A_1897216_SM5534.zip › SuppFig3.pdf]

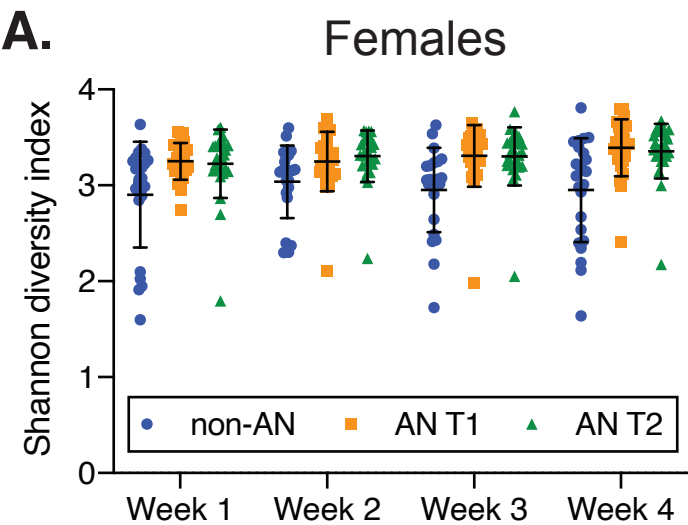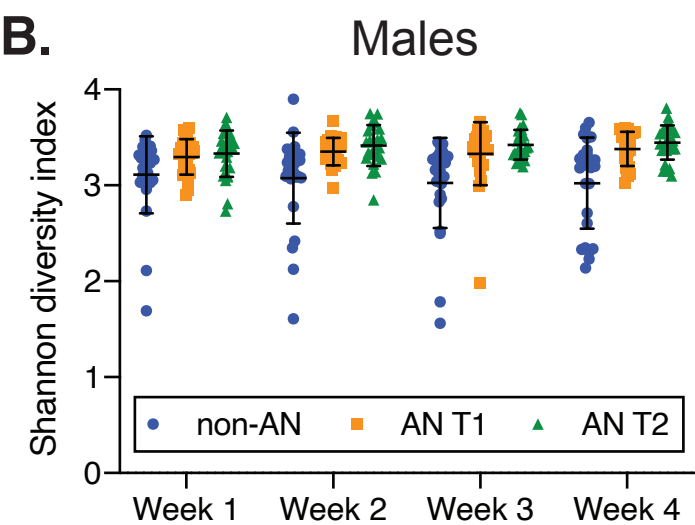

Supplement: Supplemental Material [file KGMI_A_1897216_SM5534.zip › SuppFig4.pdf]

**A.****Females**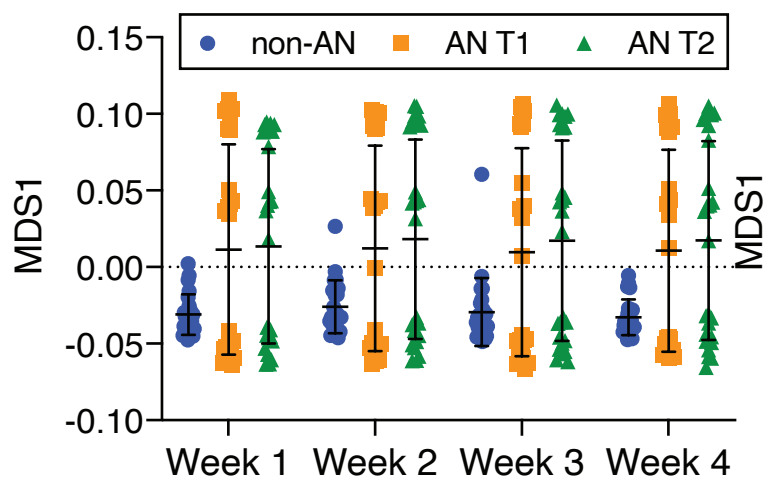**B.****Males**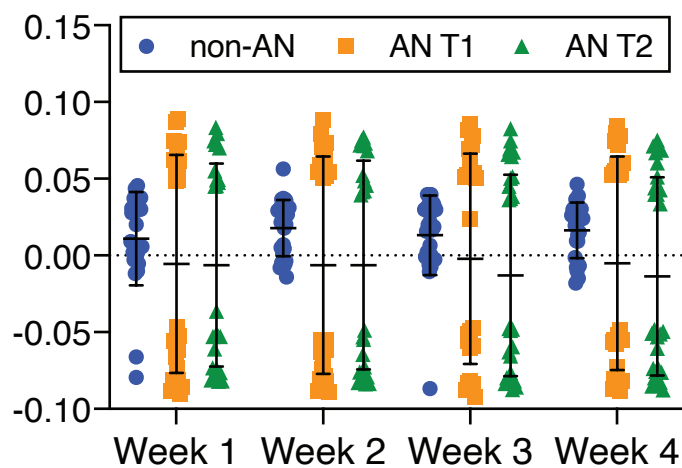**C.****Females**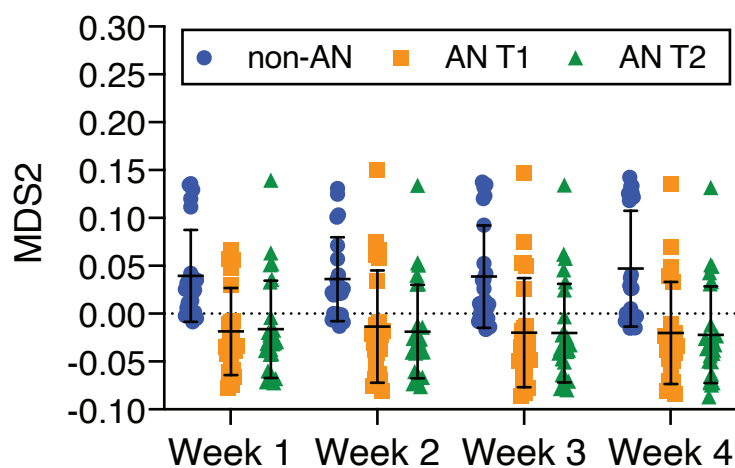**D.****Males**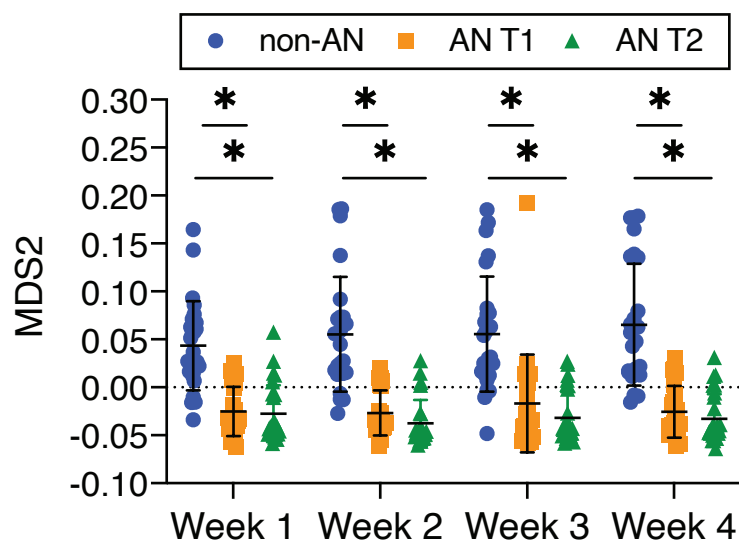**E.**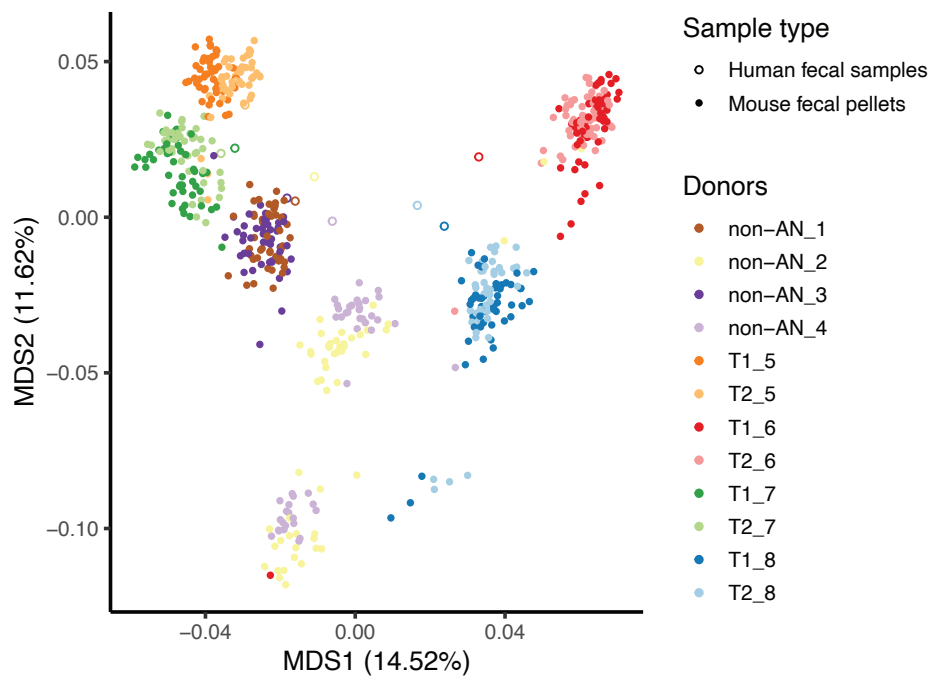

Supplement: Supplemental Material [file KGMI_A_1897216_SM5534.zip › SuppFig5.pdf]

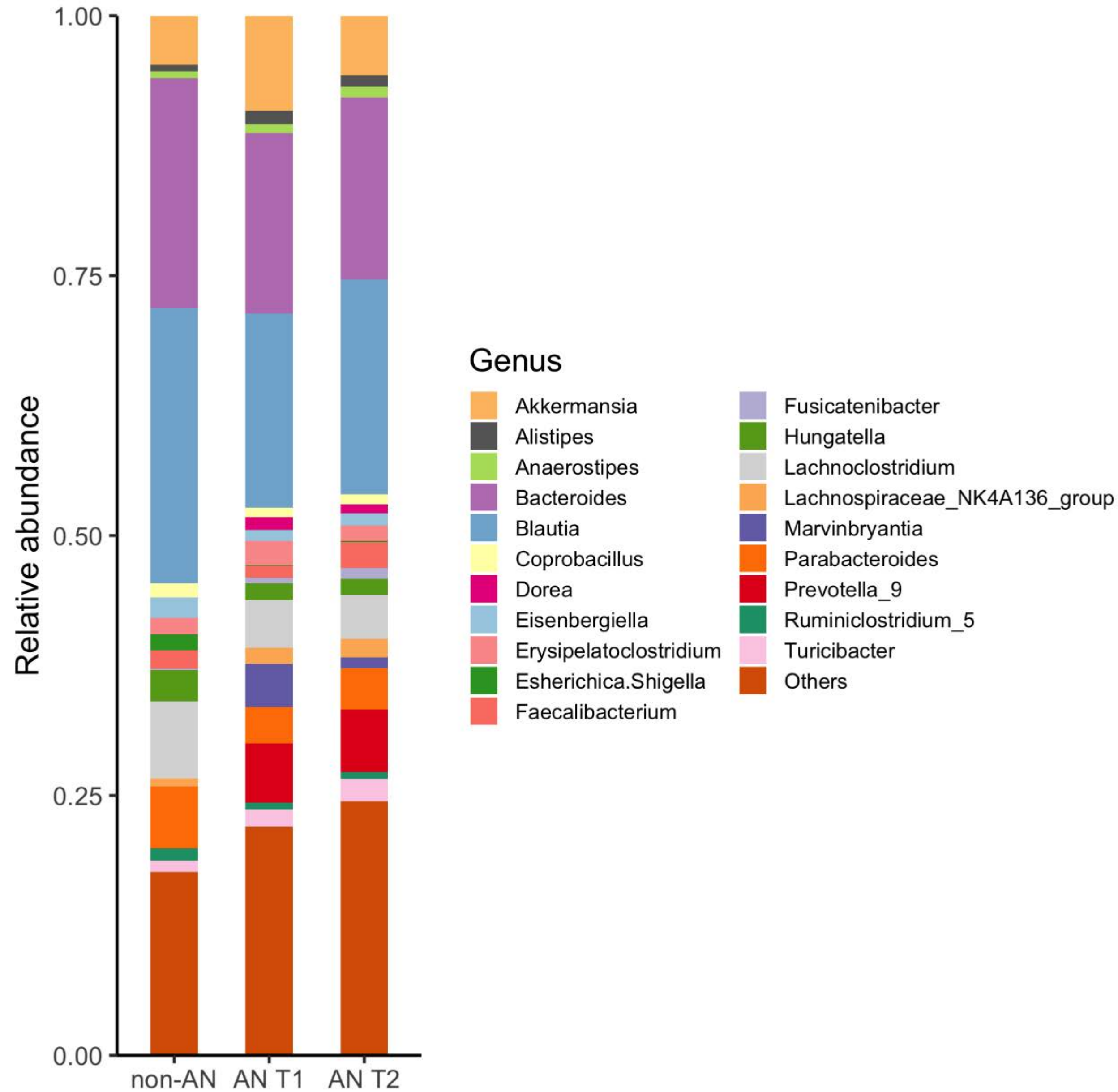

Supplement: Supplemental Material [file KGMI_A_1897216_SM5534.zip › SuppFig6.pdf]
